# Absolute Binding Free Energy Calculations for Highly Flexible Protein MDM2 and Its Inhibitors

**DOI:** 10.3390/ijms21134765

**Published:** 2020-07-04

**Authors:** Nidhi Singh, Wenjin Li

**Affiliations:** 1 Institute for Advanced Study, Shenzhen University, Shenzhen 518060, China; tanwar.nidhi7@gmail.com; 2 College of Physics and Optoelectronic Engineering, Shenzhen University, Shenzhen 518060, China

**Keywords:** binding free energy, free energy perturbation, molecular dynamics, MDM2

## Abstract

Reliable prediction of binding affinities for ligand-receptor complex has been the primary goal of a structure-based drug design process. In this respect, alchemical methods are evolving as a popular choice to predict the binding affinities for biomolecular complexes. However, the highly flexible protein-ligand systems pose a challenge to the accuracy of binding free energy calculations mostly due to insufficient sampling. Herein, integrated computational protocol combining free energy perturbation based absolute binding free energy calculation with free energy landscape method was proposed for improved prediction of binding free energy for flexible protein-ligand complexes. The proposed method is applied to the dataset of various classes of p53-MDM2 (murine double minute 2) inhibitors. The absolute binding free energy calculations for MDMX (murine double minute X) resulted in a mean absolute error value of 0.816 kcal/mol while it is 3.08 kcal/mol for MDM2, a highly flexible protein compared to MDMX. With the integration of the free energy landscape method, the mean absolute error for MDM2 is improved to 1.95 kcal/mol.

## 1. Introduction

One of the major goals of structure-based drug design is accurate prediction of protein-ligand binding affinity. The drug development process is complex and considers many factors such as high selectivity, reduced off-target activity, bioavailability of drug compounds, and toxicity effects [1]. However, the high affinity of a compound for its biological target is the prerequisite for carrying the hit compounds forward in the drug design process. Despite of the availability of lot of structural information of biological targets, the precise modelling of conformational changes of receptor upon ligand binding, entropy-enthalpy compensation, and solvent effects is a complex task [2,3]. To this end, the simulation-based computational methods have shown great promise in calculating the reliable binding free energy [4,5,6,7].

Amongst simulation-based methods, use of alchemical methods based binding free energy calculations in lead optimization and ranking of potential active compounds has shown great potential. Broadly, these methods can be approached by two ways. In the first approach, relative difference in binding energy of two different ligands for the same receptor protein is calculated. For this, one ligand is converted to another by a series of alchemical transformations in the bound state and free in solution [1,8]. This approach is better automated and widely applied. However, this approach is well suited for a congeneric series of molecules [6,9,10]. In the second approach, the standard free energy of binding can be calculated for ligand binding to receptor. For this, two sets of simulations are done in which the ligand is decoupled in the solvent and in complex with receptor [11,12]. Absolute binding free energy calculations have been performed for a few protein-ligand complexes. One of the most studied systems for absolute binding free energy calculations is the binding pocket of T4 lysozyme. In an early effort by Mobley D.L. and co-workers, absolute ligand binding energies for T4 lysozyme were calculated with root mean square error of 1.9 kcal/mol compared to experimental values [13]. Later, ligand binding affinity to relatively polar engineered cavity of T4 lysozyme (L99A/M102Q) was computed [14]. Most recently, binding energy for multiple rigid configurations for T4 lysozyme and a large set of molecules has been successfully computed [15]. Another well studied system is FK506-binding protein [16,17]. The absolute binding energy calculations have also been tested for bromodomains for the set of inhibitors [12]. Absolute binding free energy calculation is capable of comparison of binding affinities of structurally unrelated compounds. Thus, it can facilitate the early phases of drug design programs in ranking of structurally diverse compounds against the desired target.

**Figure 1:**
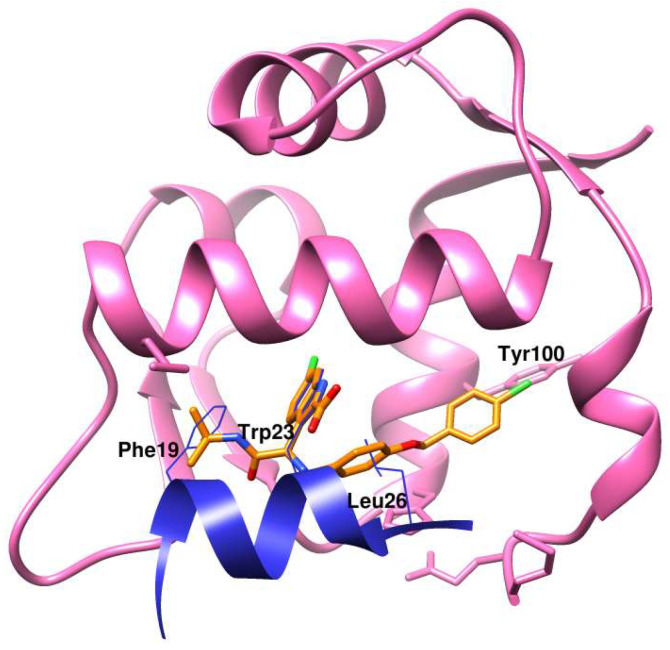
The selected target protein MDM2 in complex with transactivation domain of p53 (shown in blue color, residues Phe19, Trp23, Leu26, and Tyr100 are labelled) and ligand when bound occupies the same binding site (PDB accession code: 1YCQ and 4MDN used for representation).

In the present study, the alchemical method based absolute binding free energy calculations appended by free energy landscape analysis was proposed for a flexible protein-ligand system. To illustrate its efficacy, the proposed approach was applied to the small molecule inhibitors of p53-MDM2 interaction. MDM2 has a central role in regulation of tumor suppressor p53 activity. Thus, blocking the p53-MDM2 interaction has emerged as a promising therapeutic approach in cancer treatment [18]. The reported small molecule inhibitors have been shown to bind MDM2 mimicking the interaction between N-terminal p53 and MDM2. The triad of p53 amino acids is placed deeply into the MDM2 cleft formed by Phe19, Trp23, and Leu26 also designated as three subpockets on MDM2 [19] (Figure 1). It is noteworthy that MDM2-p53 recognition involves significant rearrangement of the Leu26 subpocket of MDM2 through twist in the Tyr100 ring referred to close and open state [20]. Similarly, induced-fit adaptation of MDM2 protein structure has been found upon binding of small molecule inhibitors [21]. Thus, MDM2-inhibitor binding involves significant structural changes in the receptor which makes it challenging to calculate accurate binding free energy [7]. In order to assess the potential of the proposed method to correlate the predicted binding free energies and reported experimental activity values for the system involving significant changes in the receptor system, this drug target was selected. Moreover, therapeutic potential of blocking the p53-MDM2 interaction and availability of inhibitors of different chemical classes makes this target suitable for absolute binding free energy calculations. In a bid to calculate true binding free energy for flexible systems, methods had been proposed earlier, mainly based upon the identification of slow degrees of freedom and regulating them deliberately while performing alchemical binding free energy calculations using techniques such as umbrella sampling [22,23]. Additionally, biased sampling using the replica exchange method have been used for enhanced sampling of protein conformations [24,25]. However, the method proposed in the present work does not require the prior knowledge of key conformational changes. Thus, the approach is general and can be applied to novel systems.

The selected target for the current study (MDM2) is highly flexible and undergoes large structural changes upon ligand binding [20,21]. The computational study carried out by Joseph et al. reported that MDM2 undergoes significant structural and dynamic changes upon ligand binding as compared to MDMX [26]. The structural differences between the holo and apo form of protein may lead to large differences in predicted binding free energy. For instance, in an earlier study of molecular dynamics based free energy perturbation (FEP/MD) for T4 lysozyme L99A mutant predicted binding free energy was overestimated due to reorientation of the side chain of residue valine upon ligand binding [27]. It is noteworthy that in an earlier study by Lee and coworkers, FEP based binding free energy calculations were done for a set of five ligands in complex with MDM2/MDMX using generalized solvent boundary potential and Spherical Solvent Boundary Potential. The study was focused on prediction of reliable near native poses of ligands. The resulted binding free energies were largely overestimated for MDM2 protein in the study due to insufficient sampling of highly flexible MDM2 protein [28]. The absolute binding free energy is dependent upon the initial conformation used for sampling. The protein structure may remain kinetically trapped in metastable state (initial conformation) and free energy cost for trapping may remain neglected. Therefore, it is proposed to generate free energy landscape of apo MDM2 which can be used to map the conformations obtained from free energy calculations onto it. The free energy difference between the energy minima apo state and the FEP calculated conformations can be used as correction term to improve the prediction of binding free energy. In the present work, FEP based absolute binding free energy calculations were performed for varied chemical classes of inhibitors against MDM2/MDMX. Subsequently, free energy landscape analysis was used for better prediction of binding energy for more flexible protein (MDM2) than MDMX. Later, prospects of MD simulation and free energy landscape analysis in sampling highly flexible apo state of MDM2 and thereof better prediction of binding free energy are discussed. In addition, use of absolute binding free energy calculations for ranking the structurally diverse compounds against the target protein are also discussed.

## 2. Results and Discussion

### 2.1. Absolute Binding Free Energy Calculations for MDMX-Ligand Systems

MDMX and MDM2 are homologous proteins and share similarity at the N-terminal region which binds to p53 protein [29]. MDMX is a non-catalytic accessory factor for Mdm2, leads to p53 protein loss, and is associated with a wide variety of human cancers. MDM2-MDMX have been reported to play non-redundant and multifaceted roles in regulating p53 activity [30]. Distinct and complementary modes of action of MDM2 and MDMX in the regulation of the pro-apoptotic activity of p53, suggests the development of dual inhibitors of the two oncogenic proteins should result in more effective antitumor strategies [29,31,32].

**Figure 2:**
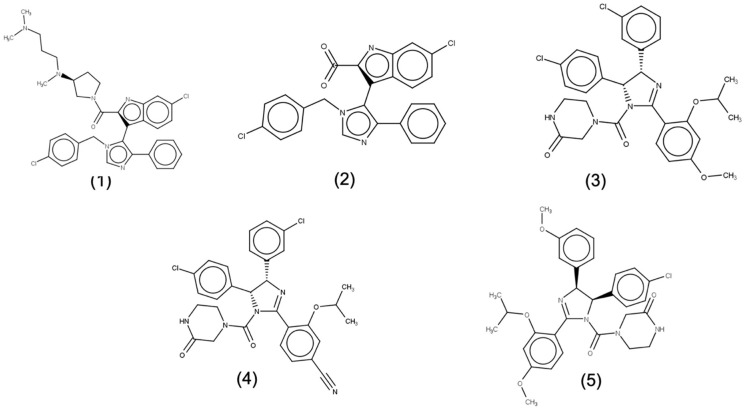
The chemical structure of ligand dataset used for FEP based binding free energy calculations for MDMX protein. The compounds are listed in Table 1.

The computational study based upon principal component analysis of molecular dynamics trajectory by Joseph et al. showed that apo-MDMX is less flexible as compared to apo-MDM2 [26]. Additionally, binding free energy calculations done by Hui Sun Lee et al. supported that MDMX undergoes minimal changes upon ligand binding [28]. In this section, absolute binding free energy calculations for five MDMX-ligand systems was completed. The crystal structures of inhibitors in complex with MDMX protein with reported binding affinity were selected. The binding affinity of selected set of inhibitors ranges from 7.0 (compound 3) to 36 µM (compound 2). The ligand dataset is shown in Figure 2. The results from this section, therefore, will indicate the efficiency of absolute binding free energy calculations in ranking of compounds according to their binding affinity. Table 1 summarizes the results obtained for this set of inhibitors in complex with MDMX protein. Firstly, the calculated binding energy values indicate a favorable binding for the compounds. Secondly, the calculated binding energy also reflects the strength of binding affinity of compounds to the target protein. The calculated binding free energy values are quite close to the experimental binding affinities calculated by ITC experiments. The mean absolute error (MAE) value for this dataset was calculated to be 0.816. The low value of MAE also indicates the high accuracy of predicted binding free energy values. The literature review suggests that absolute free energy calculation lies within 3 kcal/mol RMSD of experimental values for less flexible systems [33]. Additionally, low root mean square error (RMSE) value of 1.064 suggests that predicted values are close to experimental values. The accuracy of results obtained is comparable to previously reported free energy calculation for varied receptor-ligand complexes [12,15,34]. Thus, the results clearly indicate that binding free energy for MDMX-ligand sets were predicted with significant accuracy (Table 1).

### 2.2. Absolute Binding Free Energy Calculations for MDM2-Ligand Systems

**Figure 3:**
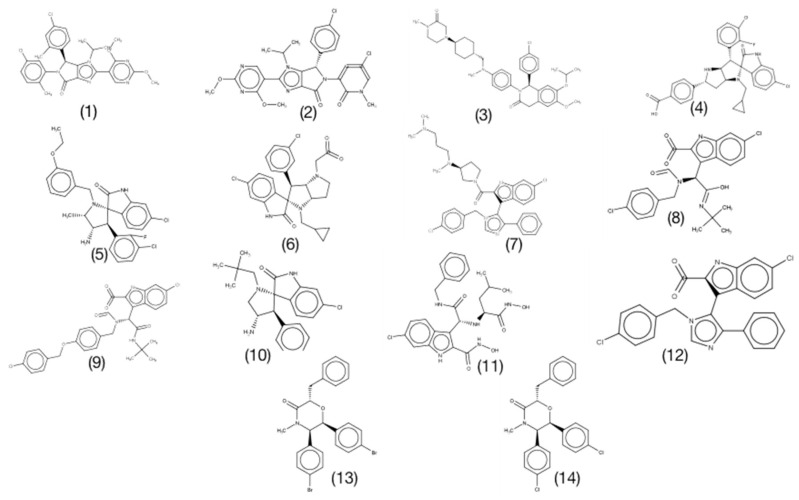
The chemical structure of ligand dataset used for FEP based binding free energy calculations for MDM2 protein. The compounds are listed in Table 2.

**Figure 4:**
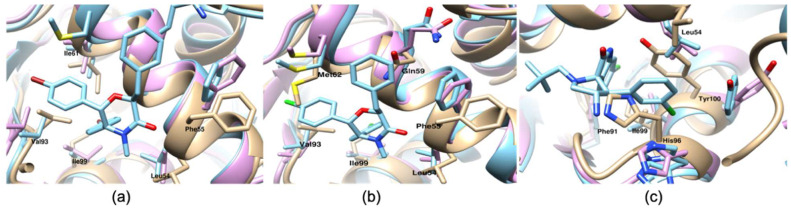
The structural comparison between apo-MDM2 crystal structure (PDB ID:1Z1M, golden color), holo-MDM2 used as start structure for free energy simulation (light blue color), and final structure obtained after simulation (hot pink color). (**a**) The protein-ligand complex (compound 13); (**b**) the protein-ligand complex (compound 14); (**c**) the protein-ligand complex (compound10). The interacting residues are highlighted and labelled in black color.

In this section, we performed binding free energy calculations for the MDM2-ligand complexes with reported binding affinity values. The selected set of inhibitors spans a broad range of binding affinity values from 0.08 (compound 1) to 1800 nM (compound 14). Additionally, the dataset represents molecules from varied chemical classes including pyrazolopyrrolidinone (compound 1), imidazolopyrrolidinone scaffold (compound 2), Dihydroisoquinolinone (compound 3), Spiro-oxindole (compounds 4, 5, 6, and 10), 6-chloroindole scaffold (compounds 9 and 11), ethyl ester of the free carboxylic acid (compound 8), imidazo-indole scaffold (compound 12), tetrasubstituted morpholinone (compounds 13 and 14) (Figure 3) which provide more reliability, meaning that the results obtained are not highly biased for a certain class of compounds. Table 2 summarizes the results obtained for this set of compounds. The predicted binding free energy (ΔG_calculated_, Table 2) was overestimated as compared to the reported experimental binding energy for all the tested protein-ligand systems. The mean absolute error value of 3.08 kcal/mol was calculated for the predicted binding free energy values. Moreover, for three out of 14 ∆complexes the calculated binding free energy was largely overestimated. For two of these complexes (13 and 14) instead a longer production run of 2 ns in each window was run. However, it did not result in improvement of predicted binding free energy (Table S2). Moreover, structural comparison between apo MDM2, ligand bound MDM2 conformation used as start structure for free energy simulations and conformation obtained post simulation was done (Figure 4). This suggests that the sampled apo-MDM2 is significantly different to the crystal structure. For instance, the side chain rotameric state of residue Phe55 is significantly different from apo crystal structure for MDM2-compound 13 complex (Figure 4a). Similarly, the side chain rotameric state of residues Phe55 and Gln59 varies significantly from apo MDM2 crystal structure for compound 14 in complex with MDM2 (Figure 4b). Notably, pi-pi interaction between benzyl ring and residue Phe55 is the key interaction in both the complexes. Additionally, for MDM2-compound 10 complex the side chain rotameric state for residue His96 varies from apo crystal structure (Figure 4c). The inadequate sampling even for single rotameric state of side chain may lead to difference of several kcal/mol in predicted binding free energy value [27]. Thus, overestimation of binding free energy values is mostly caused by insufficient sampling of apo MDM2. In order to obtain better prediction, the absolute binding free energy calculation is integrated with free energy landscape analysis method and is discussed in the next section.

### 2.3. Free Energy Landscape and Improved Prediction of Binding Free Energy

**Figure 5:**
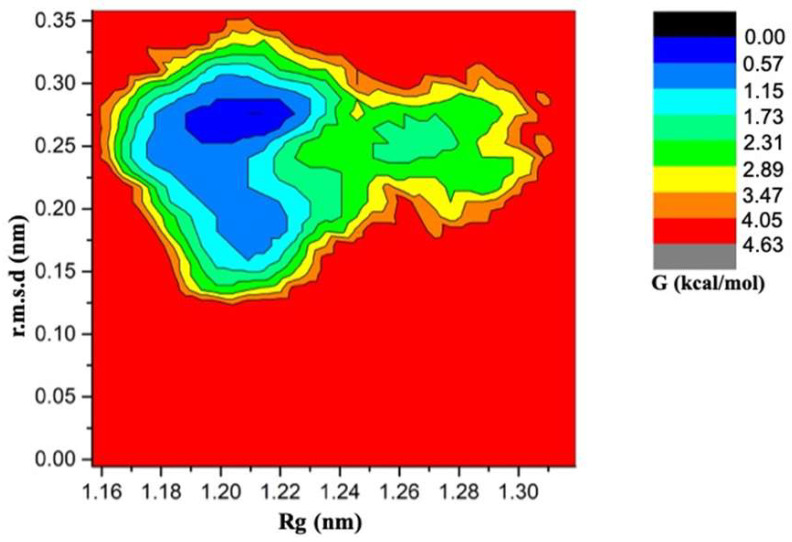
The Free Energy Landscape (FEL) between radius of gyration (X-axis) and r.m.s.d (Y-axis). The computed Gibbs free energy values are represented by colored contours.

The calculation of binding free energies is sensitive to the initial conformation of protein structure used for simulation [7,27,43]. The protein metastable states are separated by large energy barriers and the system may remain trapped in initial conformation on the simulation timescale. Additionally, insufficient sampling of even single side chain rotameric state may lead to difference of several kcal/mol in predicted binding free energies [27]. Therefore, adequate sampling of metastable states containing holo and apo state is required. The FEP simulation overestimates the binding energy because the configurations sampled in the last step of the free energy simulation may be trapped in the metastable states of the holo-state due to insufficient sampling in a rather short simulation time. Theoretically, as the transformation progresses and the interactions are turned off completely the conformations obtained in free energy simulations should represent the apo state. However, structural analysis (Figure 4) showed that the conformations obtained for sampled apo state are far from apo state MDM2 crystal structure. Therefore, sampling of apo MDM2 (PDB:1Z1M) was done and free energy landscape (FEL) was generated (Figure 5). Subsequently, FEL was used to map the conformations obtained from free energy simulation onto the generated FEL. This helped in looking at how far the free energy simulation-based conformations are from the metastable apo state. Consequently, the free energy difference between the metastable apo state and the FEP calculated conformations was used as correction term to better predict the binding free energy (Table 2). The conformations from free energy simulations were extracted from last 100 ps. A total of five conformations for each complex were extracted. The radius of gyration and root mean square deviation with respect to first frame of production run of apo state was calculated for all the extracted conformations. The free energy values corresponding to calculated Rg and r.m.s.d for extracted conformations was noted down using FEL (Table S3). The average free energy value was considered as correction term (ΔG_correction_, Table 2). The resultant binding values are indicated in ΔG_corrected_, Table 2. After applying correction term, for 11 out of 14 complexes, the predicted binding free energy lies within 2 kcal/mol of experimental values. The predicted binding free energy values showed lower mean absolute error 1.95 and root mean square error value of 2.83. The ranking correlation coefficient values were also improved (Table S4). The correction indeed improved other metrics, such as Pearson’s correlation coefficient from 0.389 to 0.430, Spearman correlation coefficient from 0.314 to 0.345. Considering that the corrections was obtained from a trivial simulation of the apo-state, such improvement is significant. In addition, scatter plot for experimental vs. corrected predicted binding energy is provided (Figure S1).

Therefore, predicted binding free energy values were found to be concordant to the experimental values. Therefore, it can be inferred that free energy landscape generation and corrected values helped in improved prediction of binding free energy for flexible MDM2-ligand systems.

### 2.4. Absolute Binding Free Energy and Drug Design

In a recent review by Williams-Noonan et al. the prospects of alchemical methods in drug design has been discussed. The study suggests that absolute free energy calculation lies within 3 kcal/mol RMSD of experimental values for less flexible and uncharged ligands and up to 4 kcal/mol RMSD for flexible and charged ligands [33]. In addition, alchemical binding free energy method even with modest level of accuracy can have considerable impact in the drug discovery process as discussed earlier by Mobley and Klimovitch [43]. For instance, in lead optimization process, the screening of ≈10–100 compounds per week with 2 kcal/mol noise can reduce the synthesis by a factor of three in order to achieve 10-fold improvement in binding affinity. However, one of the key challenges posed in reliable alchemical calculations is the substantial conformational changes incurred in the receptor [7]. The whole range of relevant protein conformations may not be accessible on the timescale of simulations. Consequently, inadequate sampling can lead to error in predicted binding free energy. Moreover, long simulations will increase the time required for free energy simulations as they are computationally expensive.

The selected target for the current study (MDM2) is highly flexible and significant structural changes occur upon ligand binding. In the light of the above facts, it can be inferred that alchemical calculations done in the current work are able to calculate binding free energy with significant accuracy. In the current study, free energy calculations resulted in more reliable prediction of binding free energy for MDM2 protein as compared to the previous study for a small dataset of five complexes and higher mean absolute error value of 7.09 kcal/mol [28]. Additionally, the calculated binding free energies are close to the experimental binding affinity. Thus, FEP based binding free energy calculations combined with free energy landscape can be used for prediction of binding free energy for flexible protein-ligand systems. Hence, ranking of compounds during lead discovery and optimization steps of drug discovery program can be done.

The alchemical calculations hold potential to provide an alternative to time consuming experimental method such as Isothermal Titration Calorimetry (ITC) [44]. Absolute binding free energies seem to be a robust and efficient method to compare the binding strength of structurally unrelated compounds, thereby addressing the challenge of scoring compounds against the target in early drug discovery steps. Furthermore, alchemical calculations can be used for prediction of selectivity of compounds against the structurally similar protein targets [45,46]. Our study also suggests that FEP based binding free energy method can be applied to discriminate the binding strength of ligands to MDM2 and MDMX proteins. Thus, alchemical calculations hold great potential not only in lead discovery and optimization but also in the field of polypharmacology. Assuming the consistent improvement in force field parameters and computational resources, alchemical calculations hold a great potential in the field of structure-based drug design.

## 3. Materials and Methods

### 3.1. Dataset

A thorough literature survey was done to collect the dataset of p53-MDM2/MDMX interaction inhibitors. In recent years, various classes of inhibitors against p53-MDM2/MDMX interaction have been reported [21,35,36,38,39,40,41,42,47,48,49,50,51,52]. The dataset was divided into two sets for further study. The first set of data consisted of inhibitors in complex with MDMX with reported experimental binding affinity values. The initial conformations of protein-inhibitor complexes were taken from the reported crystal structures (3LBJ, 2N14, 2N06, 2N0U) and ligand WK23 docked into the binding site of MDMX. The MDM2-ligand complexes with reported binding affinity values were included in the second set. Similarly, the starting conformations of protein-inhibitor complexes for the second set were fetched from reported crystal structures (6GGN, 5OC8, 4ZYF, 3TJ2, 5LAV, 5LAW, 5LAY, 5LAZ, 4JV7, 4JV9, 4MDN, 4MDQ, 3LBK) and compound WK298 docked into MDM2 binding site. This dataset comprised of varied classes of inhibitors with a broad range of inhibitory activity values.

### 3.2. Free Energy Simulations

**Figure 6:**
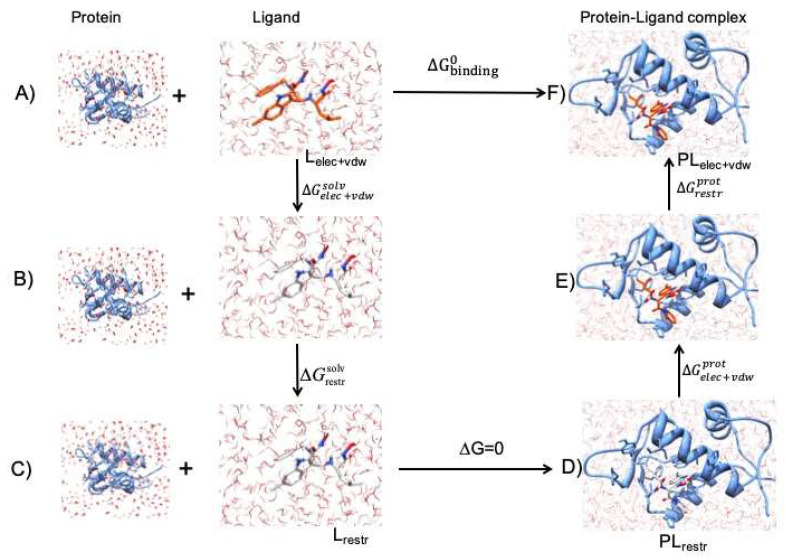
Scheme for non-physical thermodynamic cycle used to calculate absolute binding free energies, adapted from [12]. (**A**) The ligand (orange red) interacting fully with solution. (**B**) Electrostatic and van der Waals interactions turned off for ligand (light grey). (**C**) The non-interacting ligand is restrained (L_restr_), $\Delta G_{restr}^{solv}$ is calculated using the Boresch equation [11]. (**D**) Electrostatic and van der Waals interactions turned on for restrained ligand in complex with protein MDM2. (**E**) The ligand-protein complex with full interactions but restrained ligand. (**F**) Restraints removed resulting in fully interacting ligand in complex with protein MDM2. Protein and ligand were taken from PDB accession code: 4MDQ for representation of this cycle while the arrows represent the direction of cycle.

Absolute binding free energy calculations were done using the non-physical thermodynamic cycle (Figure 6). All simulations were done using GROMACS package version 2019 [53]. The initial conformations of protein-ligand complexes were taken from crystal structures (details in Section 3.1). The CHARMM36 force field was used for all the simulations. The force field parameters for ligands were generated with CGenFF server [54,55]. For each protein-ligand complex two sets of simulations were done. For the first set of simulations the ligand was decoupled from the solution. For the second set of simulations the ligand was decoupled from the complex. In both sets of simulations decoupling was done by gradually turning off coulombic and Lennard-Jones interactions. A soft-core potential was employed for transformation of the van der Waals interactions [56]. In order to decouple the ligand from solution, (A->B in Figure 6), the coulombic interactions were turned off first by changing λ from 0 to 1 with a step size of Δλ = 0.25 and the van der Waals interactions were unperturbed, then the van der Waals interactions were turned off with non-uniformly distributed values of λ (0.05 0.1 0.2 0.3 0.4 0.5 0.6 0.65 0.7 0.75 0.8 0.85 0.9 0.95 1.0). Therefore, a total of 20 windows each of 1 ns were employed for decoupling of ligand from solution. In the case of the second set of simulations (decoupling of ligand from complex), from step E to F ligand restraints were applied as bonded λ distribution (0.01 0.025 0.05 0.075 0.1 0.2 0.35 0.5 0.75 1.0) while the coulombic and van der Waals interactions were turned off in a similar manner as for decoupling of ligand in solution (D->E). Therefore, a total of 30 windows each of 1 ns were employed for the second set of simulations. The lambda points used for the transformation of the system in detail is provided (Table S1). The free energy change associated with this restraint term was calculated analytically for non-interacting ligand in solution (B->C) and corresponds to term $\Delta G_{restr}^{solv}$. On the other hand, the contribution for the interacting ligand in complex with receptor protein was calculated numerically during free energy simulations and corresponds to term $\Delta G_{restr}^{prot}$ of the thermodynamic cycle (E->F, Figure 6). For each window, 5000 energy minimization steps were carried out using the steepest descent algorithm. The system was subjected to NVT equilibration for 100 picoseconds. Temperature was coupled using Langevin dynamics [57] with reference temperature set to 298 Kelvin. Subsequently, NPT equilibration was carried out for 100 picoseconds using Parrinello-Rahman scheme. For all simulations the Particle Mesh Ewald (PME) algorithm was used to treat electrostatic interactions [58]. The LINCS constraint algorithm was used for H-bonds [59]. For production run 1 ns of simulation was carried out for each window and the data was collected. The binding free energy was calculated as sum of free energy change of formation of protein-ligand complex and the free energy of desolvating the ligand. The analytical correction term for adding restraints to decoupled ligand was also added to it. It can be expressed as:

$$\Delta G_{binding}^{o}=-\Delta G_{elec+vdw+restr}^{prot}+\Delta G_{elec+vdw}^{solv}+\Delta G_{restr\_on}^{solv}$$

The binding free energy calculations were done for all the ligand-MDM2/MDMX complexes used in this study.

### 3.3. Restraints

In order to hold the position and orientation of the ligands with respect to protein, restraint terms were used. It allowed the ligand to adopt different conformations while keeping it in the binding site such that it does not freely move out of partially interacting protein ligand system as the interactions are gradually turned off during the simulation. However, there is an entropic cost of the ligand for not allowing the free wandering throughout the simulation cell. Following the method proposed by Boresch et al. [11] the entropic cost was calculated analytically for six point restraint of the ligand in the binding site. The free energy term Δ*G_restraint_* associated with ligand restraints as interactions are turned off as:

$$\Delta G_{restraint}=RT\;ln\;\left[\frac{8\;\pi^{2}V^{o}}{\;r_{0}^{2}\sin\theta_{A,0}\;\sin\theta_{B,o}}\;\frac{\left(K_{r}\;K_{\theta A}\;K_{\theta B}\;K_{\emptyset A}K_{\emptyset B}K_{\emptyset C}\right)\frac{1}{2}}{2\pi KT^{3}}\right]$$

where *R* refers to ideal gas constant, *T* is temperature in Kelvin, *V^0^* is standard system volume for 1 molar concentration, *r_o_* is reference distance for restraints, *θ_A_* and *θ_B_* are reference angles for restraints, *K_x_* refers to strength constant of distance (*r_o_*), two angles (*θ_A_*, *θ_B_*), and three dihedrals (*ø_A_ø_B_ø_C_*). The ligands were restrained by means of one distance and force constant of 1000 kcal/mol/nm^2^, two angles, and three dihedral harmonic potentials with force constant of 10 kcal/mol/rad^2^.

### 3.4. Data Analysis

In order to calculate the free energy difference between two end states Bennett Acceptance Ratio (BAR) method was used. It calculates the ratio of weighted average of Hamiltonian difference of two given states using multiple intermediate states defined by the coupling parameter (λ) [60]. In further detail, the Hamiltonians for the states were determined by combined Hamiltonians for the end states A and B. The linear relationship H_(λ)_ = H_A_ + λ (H_B_ − H_A_); 0 ≤ λ ≤ 1 leads to a Hamiltonian representing states A and B, respectively. For instance, the free energy difference between the states *j* and *k* can be calculated as

$$\Delta G_{kj}=K_{B}T\;\left(ln\frac{f\left(H_{j}-H_{k}+C\right)k}{f\;\left(H_{k}-H_{j}+C\right)j}\right)+C$$

where *f* represents the Fermi function:

$$f\left(x\right)=\frac{1}{1+exp\left(\frac{x}{K_{B}T}\right)}$$

The *K_B_* denotes to Boltzmann constant, *T* is temperature. *H_j_* and *H_k_* represent the Hamiltonians for the states *j* and *k.* The value of C is iteratively calculated to fulfill $f\left(H_{j}-H_{k}+C\right)k=f\left(H_{k}-H_{j}+C\right)j\;$. The free energy difference is calculated as follows:

$$\Delta G_{kj}=K_{B}Tln\left(\frac{N_{k}}{N_{j}}\right)+C$$

Therefore, the free energy difference between end states *A* and *B* can be calculated as:

$$\Delta G_{BA}=\overset{n-1}{\underset{j=1}{\sum }}\Delta G_{j+1,\;j}$$

Here, *N_j_* and *N_k_* represent the number of co-ordinate frames at λ_j_ and λ_k_ states [61].

The BAR method implemented in alchemical_analysis.py python tool [62] was used to calculate free energy for two end states.

### 3.5. Free Energy Landscape Generation

The apo state of MDM2 is highly flexible and significant conformational changes are observed upon ligand binding. Therefore, in order to get thorough sampling of conformational space of apo MDM2 and thereof to estimate Gibbs free energies based on the population, two-dimensional free energy landscape was generated. The unliganded structure for MDM2 was collected from protein databank (PDBID:1Z1M) [63]. The residues 26–110 were used as start structure for 500 ns long simulation. The system was solvated in a cubic box with TIP3P water molecules at 12 Å marginal radii. At physiological pH, the structure was found to have positive charge, thus, to make the system electrically neutral, six chloride ions were added in the simulation box using the ‘genion’ tool. Then whole molecular systems were subjected to energy minimization by steepest descent minimization algorithm. Subsequently, the system was equilibrated using NVT and NPT ensemble for 100 ps each. Isotropic pressure coupling was performed using Parrinello-Rahman method. Electrostatic interactions were computed using Particle Mesh Ewald method. VdW and coulomb interactions were truncated at 1.0 nm. Conformations were stored every 10 ps. Finally, the system was subjected to production run for 500 ns. Free energy landscape was obtained by computing the joint probability distribution from radius of gyration and the root mean square deviation from the first frame of production run used as reference structure. In theory, a two-dimensional free energy landscape of the apo MDM2 is proposed to provide correction term for the bias in alchemical free energy calculations due to undersampling of the apo state when the ligand stops interacting within the binding site. The calculated root mean square deviation between the FEP calculated conformations and apo state will indicate that how far they are from each other over the free energy landscape. Subsequently, the free energy difference between apo state and FEP obtained conformations can be used as a correction term to improve the prediction of binding free energy.

## 4. Conclusions

The accuracy of absolute binding free energy is limited by conformational changes occurring in highly flexible receptor-ligand complexes. The protein conformation may remain trapped to the start structure and free energy cost associated with this trapping needs to be considered for reliable prediction of accurate binding free energy. Therefore, free energy landscape method integrated with FEP based binding free energy calculation was proposed which provides a way to correct the predicted binding free energy for flexible systems. The estimated absolute binding free energy was found to be concordant to experimentally determined values. Additionally, the proposed method resulted in the improvement of mean absolute error from 3.08 to 1.95 kcal/mol for MDM2, a highly flexible protein system. The present work features the prospect of proposed computational protocol for improved prediction of binding affinity for flexible systems. Hence, current study corroborates the use of FEP based absolute binding free energy calculations in the field of structure-based drug design.

## Figures and Tables

**Table 1:** FEP based absolute binding free energy calculations for p53-MDMX inhibitors.

S.no.	PDBID/Ligand	ΔG_calculated_ (kcal/mol)	ΔGexperimental (kcal/mol)	IC_50_ (µM)	Ref.
1	3LBJ	−6.56 ± 0.387	−6.76	11.0	[21]
2	WK23	−5.90 ± 0.341	−6.05	36.0	[21]
3	2N14	−5.95 ± 0.38	−7.3 ± 0.04	7.0	[35]
4	2N0U	−10.48 ± 0.45	−8.6 ± 0.2	8.6	[35]
5	2N06	−7.20 ± 0.38	−6.7 ± 0.2	24.9	[35]

Mean absolute error: 0.816; root mean square error: 1.064; ΔG exp was calculated using IC50 values.

**Table 2:** FEP based binding free energy calculations for p53-MDM2 inhibitors.

S. No.	Complex/PDBID	IC_50_/K_i_ (nM)	ΔG_calculated_ ^a^ (Kcal/mol)	ΔG_correction_ (Kcal/mol)	ΔG_corrected_^b^ (Kcal/mol)	∆G_experimental_ (Kcal/mol)	Ref.
1	6GGN	0.08	−17.05 ± 0.33	1.04 ± 0.07	−16.01 ± 0.4	−13.76 ^c^	[36]
2	5OC8	0.21	−14.91 ± 0.78	1.15 ± 0.08	−13.76 ± 0.86	−13.18 ^d^	[37]
3	4ZYF	1.3	−12.89 ± 0.39	1.34 ± 0.29	−11.55 ± 0.68	−12.11 ^d^	[38]
4	5LAZ	4	−12.57 ± 0.35	1.0 ± 0.03	−11.57 ± 0.38	−11.44 ^c^	[39]
5	5LAY	34	−12.04 ± 0.29	0.98 ± 0.1	−11.06 ± 0.39	−10.19 ^c^	[39]
6	5LAW	80	−11.06 ± 0.36	1.00 ± 0.03	−10.06 ± 0.39	−9.68 ^c^	[39]
7	WK298	109	−10.885 ± 0.33	1.15 ± 0.08	−9.73 ± 0.41	−9.49 ^d^	[21]
8	3TJ2	300	−11.64 ± 0.39	1.028 ± 0.09	−10.64 ± 0.48	−8.89 ^d^	[40]
9	4MDN	600	−9.79 ±0.40	1.19 ± 0.18	−8.6 ± 0.58	−8.47 ^d^	[41]
10	5LAV	819	−15.80 ± 0.25	1.15 ± 0.08	−14.65 ± 0.33	−8.29 ^c^	[39]
11	4MDQ	900	−12.52 ± 0.42	1.66 ± 0.18	−10.86 ± 0.6	−8.24 ^d^	[41]
12	3LBK	916	−10.49 ± 0.28	1.12 ± 0.03	−9.37 ± 0.31	−8.22 ^d^	[21]
13	4JV7	1000	−14.56 ± 0.29	1.42 ± 0.18	−13.14 ± 0.47	−8.63 ^c^	[42]
14	4JV9	1800	−15.73 ± 0.29	1.62 ± 0.27	−14.11 ± 0.56	−8.27 ^c^	[42]

Mean absolute error: ^a^ 3.08, ^b^ 1.95; root mean square error ^a^ 3.80, ^b^ 2.83; ^c^ ΔG_experimental_ was calculated using IC_50_ and ^d^ ΔG_experimental_ was calculated using K_i_ values. The standard error in correction term is derived from standard error of the mean (Table S3).

## Supplementary Materials

Supplementary materials can be found at https://www.mdpi.com/1422-0067/21/13/4765/s1. Table S1. The lambda points used for the transformation of system. Table S2. FEP based predicted binding free energy. Table S3. The extracted conformations for each complex and Rg, rmsd and Free Energy values. Table S4. The statistical parameter values calculated for dataset. Figure S1. Scatter Plot for Experimental Binding energy vs Predicted Binding Energy.
