# Supplementary material for: Absolute Binding Free Energy Calculations for Highly Flexible Protein MDM2 and Its Inhibitors
Source: Int J Mol Sci. 2020 Jul 4;21(13):4765. doi: 10.3390/ijms21134765 (PMC7369993; doi:10.3390/ijms21134765)
Supplement: Supplementary file 1 [file ijms-21-04765-s001.pdf]

Supplementary Information

Absolute Binding Free Energy Calculations for Highly Flexible Protein MDM2 and its inhibitors

Nidhi Singh[1,2], Wenjin Li[1,*]

1 Institute for Advanced Study, Shenzhen University, Shenzhen 518060, China

2 College of Physics and Optoelectronic Engineering, Shenzhen University, Shenzhen 518060, China

*Correspondent:

Wenjin Li, Room 341, Administration Building, Institute for Advanced Study, Shenzhen University, Shenzhen 518060, China; E-mail: liwenjin@szu.edu.cn; Tel: +86-755-26942336

S1: The lambda points used for the transformation of system

(a)For decoupling ligand from solution

coul-lambdas = 0.0 0.25 0.5 0.75 1.0 1.00 1.0 1.0 1.0 1.0 1.0 1.0 1.00 1.0 1.00 1.0 1.00 1.0 1.00 1.0

vdw-lambdas = 0.0 0.00 0.0 0.00 0.0 0.05 0.1 0.2 0.3 0.4 0.5 0.6 0.65 0.7 0.75 0.8 0.85 0.9 0.95 1.0

(b) For Decoupling ligand from protein following lambda steps were followed:

bonded-lambdas = 0.0 0.01 0.025 0.05 0.075 0.1 0.2 0.35 0.5 0.75 1.0 1.00 1.0 1.00 1.0 1.00 1.0 1.0 1.0 1.0 1.0 1.0 1.00 1.0 1.00 1.0 1.00 1.0 1.00 1.0

coul-lambdas =.0 0.00 0.000 0.00 0.000 0.0 0.0 0.00 0.0 0.00 0.0 0.25 0.5 0.75 1.0 1.00 1.0 1.0 1.0 1.0 1.0 1.0 1.00 1.0 1.00 1.0 1.00 1.0 1.00 1.0

vdw-lambdas = 0.0 0.00 0.000 0.00 0.000 0.0 0.0 0.00 0.0 0.00 0.0 0.00 0.0 0.00 0.0 0.05 0.1 0.2 0.3 0.4 0.5 0.6 0.65 0.7 0.75 0.8 0.85 0.9 0.95 1.0

S2 Table 1: FEP based predicted binding free energy

complex	Production run (1 ns for each window)	Production run (2 ns for each window)
4JV7	−14.56 ± 0.29	-14.86± 0.38
4JV9	−15.73 ± 0.29	-15.36±0.25

S3

Table 2: The extracted conformations for each complex and Rg, rmsd and Free Energy values

S.No	PDB	Rg (nm)	r.m.s.d (nm)	Free Energy (KJ/mol)	Average ± standard error of mean (KJ/mol/Kcal/mol)
1	3TJ2	1.211	0.192	3.24	
		1.220	0.187	5.66	
		1.213	0.184	4.04	4.528±0.41/1.082±0.09
		1.206	0.177	4.85	
		1.207	0.176	4.85	
2.	**4JV7**	**1.225**	**0.189**	**5.66**	
		**1.222**	**0.184**	**5.66**	
		**1.213**	**0.186**	**4.04**	**5.98±0.79/1.42±0.18**
		**1.225**	**0.180**	**5.66**	
		**1.230**	**0.190**	**8.9**	
3.	4JV9	1.220	0.166	10.5	
		1.221	0.178	8.09	

		1.223	0.181	5.66	6.79±1.13/1.62±0.27
		1.211	0.192	4.04	
		1.223	0.192	5.66	
4.	**4MDN**	**1.212**	**0.168**	**4.04**	
		**1.212**	**0.169**	**4.04**	
		**1.208**	**0.167**	**4.85**	**5.012±0.78/1.19±0.18**
		**1.213**	**0.167**	**4.04**	
		**1.222**	**0.176**	**8.09**	
5.	4MDQ	1.239	0.195	8.9	
		1.230	0.1902	8.9	
		1.222	0.1839	5.66	6.956 ± 0.79/1.66±0.18
		1.226	0.1820	5.66	
		1.228	0.1975	5.66	
6.	**5LAV**	**1.206**	**0.205**	**4.04**	
		**1.225**	**0.200**	**5.66**	
		**1.220**	**0.201**	**5.66**	**4.85±0.36/1.15±0.08**
		**1.207**	**0.207**	**4.04**	
		1.207	0.212	4.85	
7.	5LAW	1.211	0.202	4.04	
		1.204	0.213	4.04	
		1.199	0.200	4.85	4.202±0.16/1.00±0.03
		1.205	0.205	4.04	
		1.206	0.209	4.04	
8.	**5LAY**	**1.215**	**0.185**	**4.04**	
		**1.215**	**0.210**	**5.66**	
		**1.205**	**0.195**	**3.24**	**4.11±0.44/0.98±0.1**
		**1.210**	**0.198**	**3.24**	
		**1.203**	**0.186**	**4.04**	
9.	5LAZ	1.199	0.218	4.04	

		1.203	0.215	4.04	4.20±0.16/1.0±0.03
		1.206	0.206	4.85	
		1.204	0.209	4.04	
		1.205	0.208	4.04	
10.	**WK298_MDM2**	**1.223**	**0.188**	**5.66**	
		**1.205**	**0.178**	**4.85**	
		**1.215**	**0.183**	**4.04.**	**4.85±0.36/1.15±0.08**
		**1.217**	**0.173**	**5.66**	
		**1.217**	**0.198**	**4.04**	
11.	3LBK	1.219	0.206	4.04	
		1.219	0.186	4.04	
		1.214	0.189	4.04	4.68±0.16/1.11±0.03
		1.213	0.199	3.24	
		1.213	0.184	4.04	
12.	**4ZYF**	**1.216**	**0.169**	**5.66**	
		**1.220**	**0.159**	**10.5**	
		**1.216**	**0.174**	**4.04**	**5.65±1.25/1.34±0.29**
		**1.215**	**0.174**	**4.04**	
		**1.213**	**0.177**	**4.04**	
13.	5OC8	1.227	0.186	5.66	
		1.217	0.189	4.04	
		1.209	0.175	4.85	4.85±0.36/1.15±0.08
		1.227	0.180	5.66	
		1.219	0.171	4.04	
14.	**6GGN**	**1.213**	**0.183**	**4.04**	
		**1.209**	**0.186**	**4.04**	
		**1.200**	**0.189**	**4.04**	**4.36±0.32/1.04±0.07**
		**1.210**	**0.179**	**5.66**	
		**1.205**	**0.183**	**4.04**	

S4

Table 3: The statistical parameter values calculated for dataset

Dataset	Mean Absolute Error	RMSE	Pearson's Correlation coefficient	Spearman Correlation coefficient	Kendall Tau
MDMX-ligand (table 1)	.816	1.064	.860	.60	.40
MDM2-ligand (table 2, $\Delta G_{calculated}$)	3.08	3.80	.389	.314	.297
MDM2-ligand corrected set (table 2, $\Delta G_{corrected}$)	1.95	2.83	.430	.345	.297

S5: Scatter Plot for Experimental Binding energy vs Predicted Binding Energy

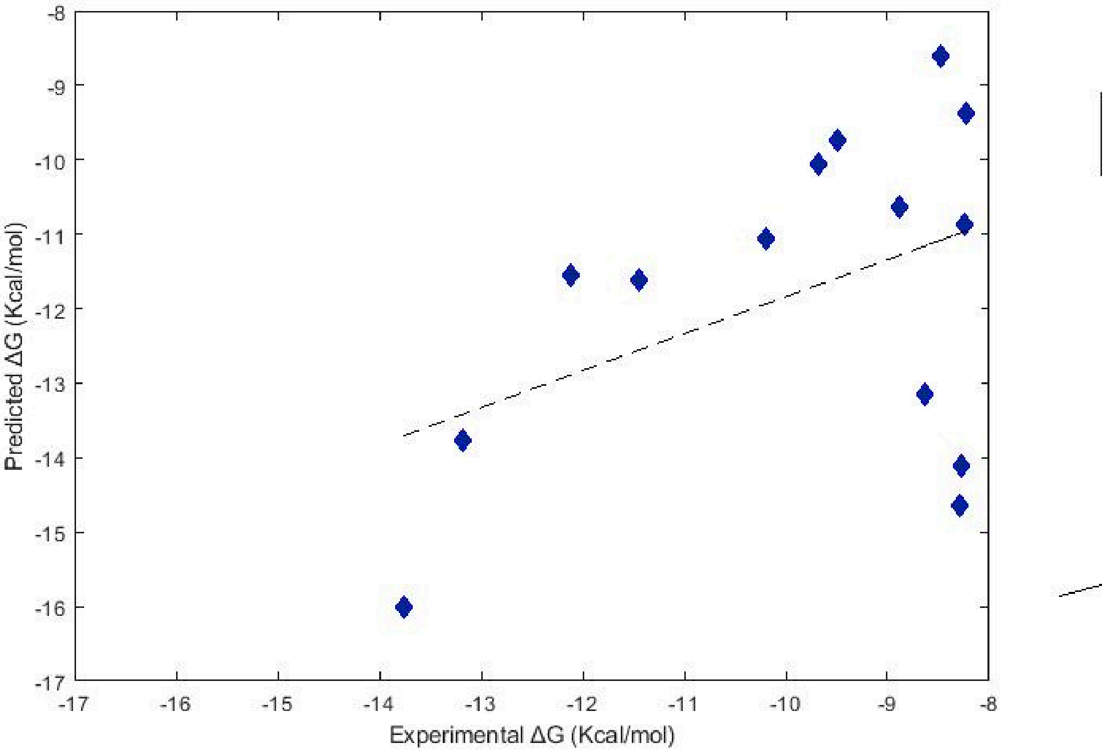

-8
-9
-10
-11
-12
-13
-14
-15
-16
-17
Predicted ΔG (Kcal/mol)
-17
-16
-15
-14
-13
-12
-11
-10
-9
-8
Experimental ΔG (Kcal/mol)
